# High-impact exercise in rats prior to and during suspension can prevent bone loss

**DOI:** 10.1590/1414-431X20155086

**Published:** 2016-02-02

**Authors:** G.R. Yanagihara, A.G. Paiva, G.A. Gasparini, A.P. Macedo, P.D. Frighetto, J.B. Volpon, A.C. Shimano

**Affiliations:** 1 Laboratório de Bioengenharia, Departamento de Biomecânica, Medicina e Reabilitação do Aparelho Locomotor, Faculdade de Medicina de Ribeirão Preto, Universidade de São Paulo, Ribeirão Preto, SP, Brasil; 2 Instituto Federal de Educação, Ciência e Tecnologia de São Paulo, São Paulo, SP, Brasil

**Keywords:** Bone, Mechanical properties, Bone mineral density, Scanning electron microscopy, Jump, Rats

## Abstract

High-impact exercise has been considered an important method for treating bone loss in osteopenic experimental models. In this study, we investigated the effects of osteopenia caused by inactivity in femora and tibiae of rats subjected to jump training using the rat tail suspension model. Eight-week-old female Wistar rats were divided into five groups (n=10 each group): jump training for 2 weeks before suspension and training during 3 weeks of suspension; jump training for 2 weeks before suspension; jump training only during suspension; suspension without any training; and a control group. The exercise protocol consisted of 20 jumps/day, 5 days/week, with a jump height of 40 cm. The bone mineral density of the femora and tibiae was measured by double energy X-ray absorptiometry and the same bones were evaluated by mechanical tests. Bone microarchitecture was evaluated by scanning electron microscopy. One-way ANOVA was used to compare groups. Significance was determined as P<0.05. Regarding bone mineral density, mechanical properties and bone microarchitecture, the beneficial effects were greater in the bones of animals subjected to pre-suspension training and subsequently to training during suspension, compared with the bones of animals subjected to pre-suspension training or to training during suspension. Our results indicate that a period of high impact exercise prior to tail suspension in rats can prevent the installation of osteopenia if there is also training during the tail suspension.

## Introduction

Osteoporotic fractures represent an important public health problem worldwide (1 2 3). Currently, it is estimated that 30 to 50% of women and 15 to 30% of men will suffer an osteoporotic fracture at some point in their life (1). In the United States, about 10 million Americans over the age of 50 have osteoporosis, while another 34 million have osteopenia. These conditions can lead to a diminished quality of life and increased risk of death. In Brazil, high rates of osteoporotic fractures have been reported by BRAZOS (Brazilian Osteoporosis Study) (4,5). The World Health Organization defines osteoporosis as the changes in bone micro-architecture resulting in a progressive loss of bone matrix quality, and decrease in the physical properties of the bone, such as bone mineral density (BMD) and elasticity (6,7). Osteopenia is a reversible condition that precedes osteoporosis (7).

Several factors are helpful in the fight against bone loss, including a healthy life style, no smoking, use of therapeutic drugs and regular physical exercise (8,9). The mechanical stress caused by physical activities is crucial for the structural and functional integrity of the skeletal system, since it increases bone density and mechanical strength (1,). For this reason, physical exercise has been referred as both a preventive and a therapeutic activity against bone loss and muscle atrophy. The increase of BMD (1,2,9), and bone mass and strength (10,16,17) by physical exercise have been shown in human and animal studies. One of the most effective physical activities in eliciting osteogenic response is the high impact exercise, as it causes a high tension and deformation rate in the bones (1,13 [Bibr B14]). Thus, jumping appears to promote superior bone formation compared with aerobic exercises (11 [Bibr B12],^18^ ^19^ ^20^ ^21^).

The rat tail suspension test is an efficient model for inducing osteopenic changes secondary to physical hypoactivity (22 23 24). Some studies have used this model to investigate the effects of the jump exercise during the tail suspension period, in preventing the occurrence of osteopenia (11,18,25). Furthermore, the jumping exercise during a remobilization period was evaluated as a treatment for previously established osteopenia (25). However, studies investigating jumping exercises before tail suspension and its effects in bone quality and the onset of osteopenia, were not found in the literature.

Therefore, the aim of this study was to evaluate whether training with jumps before and/or during the period of tail suspension of rats can prevent or minimize the bone loss caused by hypoactivity.

## Material and Methods

This study was approved by the Ethics Committee on Animal Experimentation of the Faculdade de Medicina de Ribeirão Preto, Universidade de São Paulo, Brazil (#2013/085). Procedures were performed in accordance with the International Guiding Principles for Biomedical Research Involving Animals (26) and with the Brazilian College of Animal Experimentation.

### Animals

Eight-week-old female Wistar rats (190-210 g) were supplied by the vivarium of the Faculdade de Medicina de Ribeirão Preto, Universidade de São Paulo. Rats were housed in specific cages in a quiet environment and under normal conditions of controlled temperature (22±2°C) and humidity (55-60%). A 12-h light/dark cycle was maintained, and animals had access to standard rat chow and water *ad libitum*. Body weight was measured once a week. At the end of the experiment the rats were sacrificed with an overdose of ketamine chlorhydrate and xylazine chlorhydrate. Both femora and tibiae were then collected, cleaned of soft tissue and kept in a freezer.

### Experimental design

**Figure 1:**
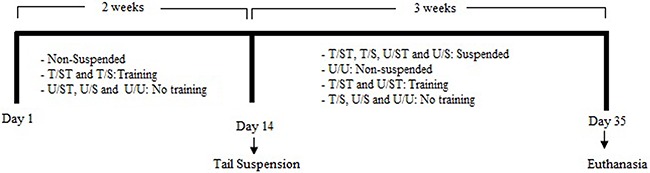
Experimental design. Rats were divided into 5 groups (n=10) as follows: T/ST: jump training before suspension and training during suspension; T/S: jump training before suspension; U/ST: training only during suspension; U/S: suspension without any training, and U/U: untrained and unrestricted in regular cages.

The rats were divided into 5 groups (n=10 per group), as follows: T/ST: jump training before suspension and training during suspension; T/S: jump training before suspension; U/ST: training only during suspension; U/S: suspension without any training, and U/U: untrained and unrestricted in regular cages. The experimental design is shown in Figure 1.

### Tail suspension protocol

**Figure 2:**
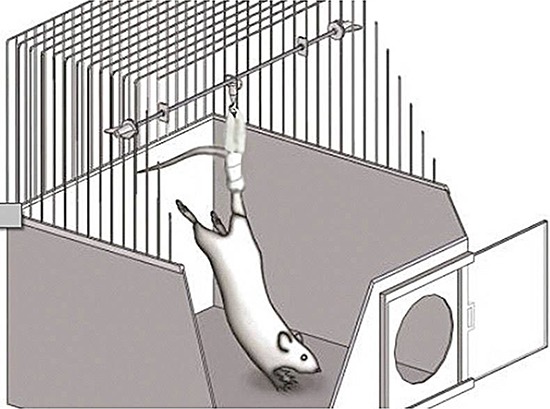
Schematic drawing of the rat tail suspension model.

The tail suspension procedure was performed in accordance with the recommendations by Holton and Globus (23). A strip was wrapped around the tail at 2 cm from its base, leaving its distal third free for circulation monitoring. The strip formed a loop to be connected to a swivel, which was connected to a transversal bar. The overhead trolley system allowed 360° of rotation and was adjusted to keep the rat trunk at 30° of inclination, thus permitting the animal to move around to reach for food and water, but not touching the cage floor or walls with the hind limbs (Figure 2). The rats were kept in suspension for 3 weeks. The rats submitted to training during the suspension period were removed from the suspension, submitted to jumping exercise and returned to the suspension thereafter.

### Jumping exercise protocol

The jump training was performed by placing the rat inside a wooden box (40×40×40 cm; Insight^¯^, Brazil). To elicit the first jumps the animals received an electrical stimulation delivered by a metal grid covering the box internal floor. The electrical discharge was controlled by the researcher. With the stimulus, the animal jumped and grasped the upper edge of the box with the forelimbs and climbed onto a board. The rat was then returned to the bottom of the box to repeat the procedure. After about 1 week the rats learned to jump as soon as they were placed in the box, so that the electrical stimulus was no longer necessary. The training protocol consisted of 20 jumps/day for 5 days a week. The training period before suspension had a duration of 2 weeks, and during suspension, 3 weeks. The initial jump height was 25 cm, which was gradually increased to 40 cm by the end of the first week (11,18).

### Bone densitometry

The BMD of the left femora and tibiae was measured by a dual-energy X-ray absorptiometry densitometer (DXA; Hologic QDR^¯^, Discovery™, USA), with special QDR^¯^ software for small animals, with high scanning resolution, following the proximal-distal direction. The analysis was performed in the femoral head, neck and trochanteric region and in the whole tibiae.

### Mechanical analysis

**Figure 3:**
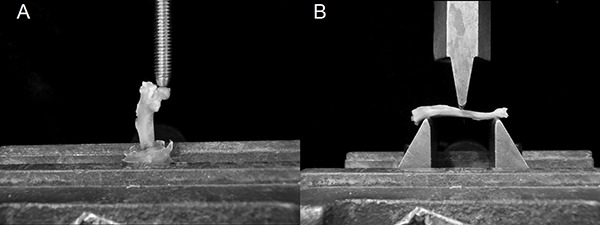
Mechanical testing. *A*, flexo-compression test of the femora and *B*, 3-point bending flexural test of the tibiae.

The mechanical properties of maximum load (L_max_) and stiffness (St) were determined by testing left bones to fracture in an universal testing machine (EMIC^¯^, DL 10000, Brazil) equipped with a load cell of 500 N, and TESC software, version 13.4 (EMIC). For the left femur, flexo-compression force was vertically applied to the femur head at a speed of 0.5 mm/min. Differently, the tibiae were tested by the three-point bending flexural test, with force applied at a speed of 1.0 mm/min in the antero-posterior direction (Figure 3). In both tests, a 5 N preload was used for 30 s.

### Scanning electron microscopy

**Figure 4:**
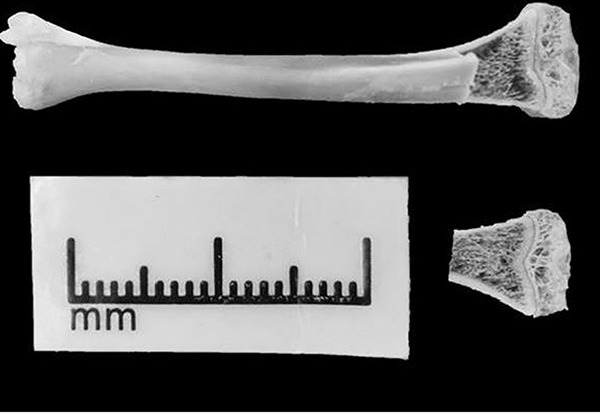
Preparation and analysis by scanning electron microscopy.

For the analysis by scanning electron microscopy (SEM), the right tibiae were kept in absolute ethyl alcohol for 5 days then cut (1-mm thick) in the coronal plane using an ISOMET^¯^ 1000 saw (Buehler, USA) (Figure 4). After cutting, the slices were attached to aluminum supports (stubs) with silver- and graphite-based conductive glue to improve the flow of electrons. The stubs were kept dry with silica gel (Synth^¯^ Labsynth, Brazil) and subsequently sputter-coated in gold, in a SCD 050 Sputter Coater (Bal-Tec™, Germany), for 350 s. The stubs with the samples were then positioned in an Evo-MA10 electron microscope (Zeiss™, Germany). The images were obtained with 40× magnification and the trabecular spaces were analyzed qualitatively.

### Statistical analyses

Statistical analyses were performed using SPSS 16.0 for Windows (IBM, USA). Normality of the data was tested with the Shapiro-Wilk test. Results were compared using ANOVA with the Tukey's *post ho*c test (parametric data) or Kruskal-Wallis with Dunn's *post hoc* test (non-parametric data). The differences are reported as percentages. P≤0.05 was considered to be statistically significant.

## Results

### Body mass

At the beginning of the study, body mass was similar among the groups. After 4 weeks, the control group (U/U) had greater body mass than all suspended rats (9.72% higher than T/ST, 14.02% higher than T/S, and 20.85% higher than U/S, P<0.001). The suspended group without training (U/S) had 9.21% lower body mass than T/ST, and 15.44% lower than U/ST (P<0.001). At the end of the experiment the groups submitted to training (T/S, T/ST and U/ST) had body weight similar to the control group (U/U). The control group body weight was 13.06% higher than the suspended group without training (U/S) (P<0.02) (Table 1).

**Table:**
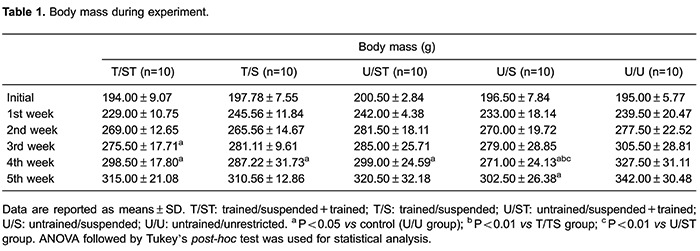

### BMD

*Femora*. Multivariate comparison showed significant differences in BMD of the femora between groups (P<0.001; η_p_^2^=0.725). The femora BMD of animals from T/S group was 12.05% lower than U/U, 10.83% lower than T/ST, and 10.07% lower than U/ST (P<0.01). The animals of the U/S group had BMD 16.66% lower than those of U/U, 15.49% lower than T/ST, and 14.78% lower than U/ST (P<0.01). There was no significant difference in BMD of the femora of the T/ST and U/ST groups compared to the control group (P>0.05) .

**Figure 5:**
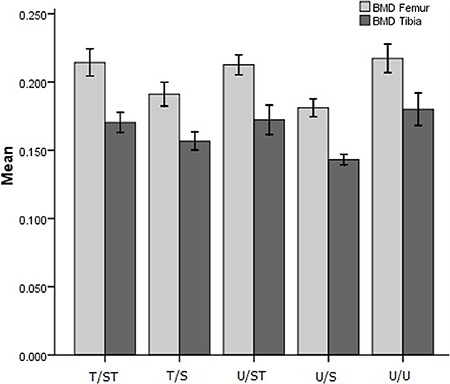
BMD (bone mineral density; g/cm^2^) of the experimental groups. T/ST: trained/suspended+trained rats; T/S: trained/suspended rats; U/ST: untrained/suspended+trained rats; U/S: untrained/suspended rats; U/U: untrained/unrestricted. Results are reported as mean and 95%CI for n=10 rats per group.

*Tibiae*. Multivariate comparison showed significant differences in tibiae BMD between groups (P<0.0001; η_p_^2^=0.725). The animals of group T/S had tibiae BMD 12.89% lower than U/U, 7.98% lower than T/ST, and 9.00% lower than U/ST (P<0.01). The animals of the U/S group had tibiae BMD 20.50% lower than U/U, 16.02% lower than T/ST, and 16.95% lower than U/ST (P<0.01). There was no significant difference in tibiae BMD of the animals from the T/ST and U/ST groups compared to the control group (P>0.05; Figure 5).

### Mechanical properties

*Femora*. Multivariate comparison showed significant differences for L_max_ of the femora between groups (P<0.001, η_p_^2^=0.525). The T/S group had L_max_ of femora 28.32% lower than U/U, and 29.02% lower than T/ST (P<0.01). The U/S group had L_max_ of femora 24.44% lower than U/U, and 24.96% lower than T/ST (P<0.01). The L_max_ of femora of the T/ST and U/ST groups were not statistically different to those of the control group (P>0.05). The St of the femora was not significantly different between groups (P>0.05, η_p_^2^=0.118).

**Figure 6:**
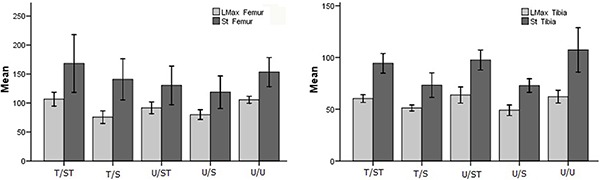
Mechanical properties of the femora (*left panel*) and tibiae (*right panel*). T/ST: trained/suspended+trained rats; T/S: trained/suspended rats; U/ST: untrained/suspended+trained rats; U/S: untrained/suspended rats; U/U: untrained/unrestricted. L_max_: maximum load (N); St: stiffness (N/mm). Results are reported as mean and 95%CI for n=10 rats per group.

*Tibiae*. Multivariate comparison showed significant differences for L_max_ of the tibiae between the groups (P<0.001, η_p_^2^=0.548). The animals of T/S had L_max_ 17.54% lower than U/U, 15.04% lower than T/ST, and 19.53% lower than U/ST (P<0.001). The animals of U/S had L_max_ 25.28% lower than U/U, 22.91% lower than T/ST and 26.99% lower than U/ST (P<0.001). The L_max_ and St of tibiae of the T/ST and U/ST groups were not statistically different to those of the control group (P>0.05). Multivariate comparison showed significant differences for St of the tibiae between groups (P<0.001, η_p_^2^=0.438). The tibiae St was 31.73% lower in the animals of the T/S group than U/U, 22.34% lower than T/ST and 24.90% lower than U/ST (P<0.01). The tibiae St of the U/S group was 25.28% lower than U/U, 22.72% lower than T/ST, and 25.27% lower than U/ST (P<0.01) (Figure 6).

### SEM

**Figure 7:**
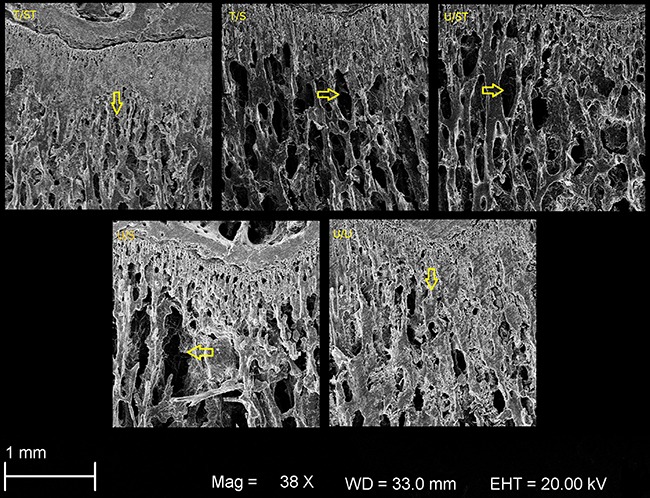
Scanning electron microscopy showing comparative bone micro-architecture. Arrows indicate trabecular space. T/ST: trained/suspended+trained rats; T/S: trained/suspended rats; U/ST: untrained/suspended+trained rats; U/S: untrained/suspended rats; U/U: untrained/unrestricted.

Qualitative analysis of the SEM image showed that the T/ST and U/U groups had smaller spaces between trabeculae, indicating a higher bone microarchitecture quality compared with T/S, U/ST and U/S. The animals that were only suspended but not trained (U/S group) had the lowest bone microstructural quality (Figure 7).

## Discussion

The main finding of this study was that the animals subjected to jumping exercises before and during tail suspension resulted in an overall higher bone quality when compared with animals, which were submitted to jumping either before or during suspension.

In 1979, to simulate bone changes induced by microgravity as experienced by astronauts or animals in space flights, Morey ER (27) developed a model in which the hind limbs of rodents were suspended by the tail. This model, now a well-accepted method to evaluate osteopenia caused by disuse/hypoactivity (11,18,25,28), allows the development of studies that would not be feasible in human subjects, specially because of ethical aspects and the required long follow-up. Loss of body mass is an important indicator of stress in this model. According to Morey-Holton and Globus (23), a limit of 10% weight loss in 24 h is acceptable, which must be recovered within 2 days. In our study, body mass increased each week in all groups, which is consistent with the recommendations by Morey-Holton and Globus (23). Suspended rats had a smaller and slower body mass gain than unrestricted rats. Furthermore, we observed that jump training prevented loss of body mass. These findings are in agreement with other studies (18,23,29,30).

Physical training is known to counteract osteopenia caused by disuse (8,18,31). The positive effects of exercise on BMD, added to the other known benefits of exercise, such as a balanced and improved muscle function, can reduce the incidence of fractures by up to 50% (31,32). In addition, training with jumps has been shown to be effective for treatment as well as prevention of bone osteopenia in rats (13,18,25,).

Continuous training seems to be necessary for maintaining bone quality (36 36 37). However, there are reports in which the beneficial effects promoted by high-impact training can be maintained after a non-training period of up to 6 months (10,38). The inclusion of a training session before tail suspension in the current study was based on the hypothesis that the acquired benefits could minimize the deleterious effects caused by hypoactivity.

We found that tail suspension caused deterioration in bone quality, which is in accordance with several authors (11,18,25) who found a decrease of mechanical properties such as stiffness, resilience and maximum load limit in adult rats subjected to tail suspension. On the other hand, training with jumps improved BMD and femoral strength. Similar results were found in the studies by Ju et al. in 2012 (11) and 2013 (18), in which the suspended rats had decreased bone mass, and suspended rats trained with jumping exercises showed bone mass similar to those of the control group. However, in these studies rats did not have any exercise prior to suspension. In the current study, the positive findings in bone quality of the animals trained only during suspension (U/ST) compared to the suspended group that never trained (U/S) suggest that this protocol might be also effective in preventing osteopenia.

Although many studies use BMD as a measure of bone fragility, currently it has been reported that up to 80% of low-trauma fractures occur in individuals without osteoporosis (1,33), indicating that bone densitometry by DXA may not be sufficient to obtain an accurate measure of bone strength. Another disadvantage of bone densitometry, is the variation in bone mass and geometry depending on the area analyzed. A larger cross section of the bone can lead to a higher measure of bone strength, without real changes in BMD. Furthermore, trabecular micro-architecture of bones can adapt to force loading, but most imaging techniques are still limited in their measuring accuracy to detect these changes. For these reasons, the effects of physical exercise on bone micro-architecture are not yet fully understood (1). As far as we know, this is the first study in this line of research that used SEM analysis.

The results found for BMD and mechanical analysis are consistent with each other and confirmed by electron microscopy. The trabecular space was larger in suspended animals compared to control, and smaller in trained animals compared to non-trained animals, supporting the positive results of exercise training before and during tail suspension. Ju et al. in 2013 (18) observed architectural deterioration in the trabecular network of the femora mainly attributed to the reduced number of trabeculae in suspended rats, assessed by computerized microtomography. This deterioration was not found in animals trained with jumping exercises.

The duration of the jump training protocol reported in the literature varies: 24 weeks (39), 8 weeks (10,33),[Bibr B34] 5 weeks (25), 3 weeks (18), and 1 week (15). In our study, a total training period of 5 weeks was set, with 2 weeks of pre-suspension training and 3 weeks of training during suspension.

Although pre-suspension training alone demonstrated higher values than absence of training, the difference was non-significant. This suggests that a longer period of pre-suspension training by itself might generate positive effects on bone after suspension. Therefore, further studies with different exercise protocols, especially regarding duration, are necessary to better understand the effects of physical training followed by a period of hypoactivity.

In conclusion, our findings showed that tail suspension reduced BMD and mechanical properties of the femora and tibiae of rats, and increased trabecular space. Pre-suspension jump training alone was not effective to prevent bone loss due to disuse, but jump training prior to and during tail suspension was beneficial to the bone, keeping the physical properties unchanged.
